# Doxycycline sclerotherapy as a primary treatment of head and neck giant cystic hygroma: A case report study

**DOI:** 10.1016/j.ijscr.2025.110945

**Published:** 2025-01-25

**Authors:** Waddah Al-Saadie

**Affiliations:** Otolaryngology Department, Damascus Hospital, Damascus, Syria

**Keywords:** Case report, Doxycycline, Sclerotherapy, Cystic hygroma

## Abstract

**Introduction:**

Cystic hygroma is a benign lymphatic malformation, developing around 6th gestational week. The big challenge is in the way they are managed due to their extension within the vital elements. Treatment options include watchful waiting, surgery, sclerotherapy and combination between them in some cases.

**Case presentation:**

We present a rare case of giant cystic hygroma of a 11-month-old male was referred to the Department of Otolaryngology for an asymptomatic, aesthetically unappealing swelling in the neck since 9 months. A well-defined large cystic mass measures about (4 × 7 × 9 cm) was seen in the CT scan, originating at the left lateral neck. It extends from the left submandibular space inferiorly posterior to the sternocleidomastoid muscle, and retrosternally to the aortic arch level. From the Posterior aspect, the mass reaches the prevertebral space. No lymph adenopathy was noted. No bony erosions. Findings are suggestive of Cystic Higroma (CH).The primary treatment was sclerotherapy using doxycycline for one time. The procedure was performed at an operating theatre under general anesthesia because injection of the sclerosant factor is painful. The child was monitored for 3 months after the procedure (with an interval of one month between each observation). There was a noticeable improvement after 4 weeks and the complete resolution of the cystic hygroma was observed 12 weeks after the initial procedure. During the monitoring period of 6 months, the lesion did not show any recurrence.

**Clinical discussion:**

In this case of giant Cystic Higroma (CH) significantly decreased after using of Doxycycline sclerotherapy for one time only without the need to repeat the procedure more than once. So clinicians should be aware of this good treatment of (CH) because it offers minimal patient trauma and excellent outcomes.

**Conclusion:**

Through the good results we have reached from this case, we encourage its application extensively in the future to more cases due to their safety and quick results.

## Highlights

• Nowadays, surgical excision has become the less popular option for CH with high rates of complication and it has been successfully replaced with intralesional sclerotherapy as approved by many previous studies.
• Our preliminary results with doxycycline sclerotherapy as an alternative form of treatment for cervical cystic hygromas are encouraging.
• Adequate diagnostic workup, classification and staging of cystic lymphangiomas are paramount for proper individualized treatment selection.
• The traditional absolute surgical option employed by developing countries need to be revised as the astounding recurrence rates and potential surgical morbidity can be avoided in some patients.
• We injected an amount of Sclerosing material equal to half the amount withdrawn from the cyst.
• we obtained a result similar to the results obtained in studies in which injections were used several times within certain intervals according to each study.

## 1 Introduction

Cystic hygroma (CH) is considered a relatively rare lesion, but it is one of the most common benign congenital Lymphatic malformations. It was described first by Redenbacher in 1828 [1].

Approximately 80 % of (CH) occur in the cervicofacial region but it could affect any part of the body [1]. CH usually develops around 6th gestational week, because of embryonic occlusive jugular-lymphatic channels or abnormally sequestered lymphatics.

The incidence of this lesion is 1 in 2000–4000 live births [2]. The disease is visible at birth in about half of the cases, but in 80 % to 90 % of cases are diagnosed during the first or second year of life.

It has life-threatening complications, including respiratory obstruction, dysphagia, nerve compression, and malocclusion. In embryonic life if a (CH) appears in the neck increases the risk for airway obstruction and it requires intrapartum fetal intervention [3].

There are multiple ways to treat (CH). Previously, surgery was considered the primary treatment, but the total excision has a high risk to the patient because it can lead to severe complications such as neurovascular injury and cosmetic deformity. Fortunately, that large cysts respond very well to percutaneous sclerotherapy with less surgical morbidity [4,5].

Several classification systems have been proposed to aid in diagnosing and managing cystic hygromas. De Serres et al. proposed a classification system based on the anatomic location of the lymphatic malformation in relation to the hyoid (it is allowed for prognostication and estimation of the surgical complication rate) [6]. Smith and colleagues described the cystic lymphangiomas as either macrocystic, microcystic, or mixed, depending on radiological findings. Macrocysts were defined as lesions with cystic contents of at least 2 cm3 in volume, whereas microcysts contained lesions less than 2 cm3. Furthermore, this classification is also capable of predicting the response to sclerotherapy [7].

Doxycycline is a derivative of tetracycline and is a broad-spectrum antibiotic.

We chose Doxycycline in this case as the sclerosing agent because it is widely available, relatively inexpensive, theoretically may prevent infectious complications and it has an established safety profile.

In our case, we aimed to focus on the management of a 11-month-old male patient who referred to the Department of Otolaryngology with a large cervical cystic hygroma which was successfully treated with doxycycline sclerotherapy for one time only.

This case is described in accordance with the criteria of SCARE [8].

## 2 Presentation of case

### 2.1 Patient information

We present the case of a 11-month-old male patient who referred to the Department of Otolaryngology for an asymptomatic, aesthetically unappealing swelling in the neck.

The mass appeared at the age of two months and gradually increased in size. There is no local pain, fever, respiratory distress, dysphagia or weight loss. But there is a hoarseness when the child cries. The patient had not undergone any previous medical or surgical intervention. There is no Drug history or family history including any relevant genetic information.

During the last period, the child's family visited a general practitioner and the case was not diagnosed and no therapeutic measures were taken.

## 3 Clinical findings

**Fig. 1:**
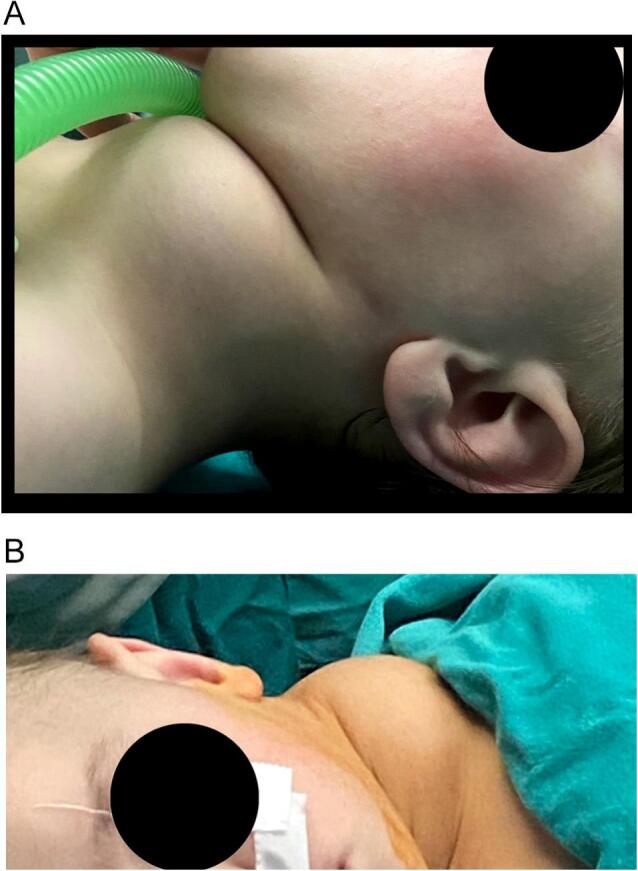
A: Clinical image showing the extent of swelling in the patient's left neck area before surgery (lateral view). B: Clinical image showing the extent of swelling in the patient's left neck area before surgery (frontal view).

Upon arrival, the patient was seen in the clinic where the general condition was good, with noticeable swelling extends from the left submandibular space inferiorly extended to the chest (Fig. 1A–B) with significant difficulty breathing. This complaint appeared 6 months ago and gradually increased until the child visited the clinic.

On clinical examination:

A (6 × 5 cm) nontender cervical neck mass involving both the left anterior and posterior triangles was evident. The neck mass was smooth, mildly compressible, and not attached to the skin. There were neither palpable lymph nodes in the neck nor any other swellings elsewhere and Intraoral examination and flexible nasolaryngoscopy is normal.

## 4 Diagnostic assessment

**Laboratory tests** where within normal limits.

**Fig. 2:**
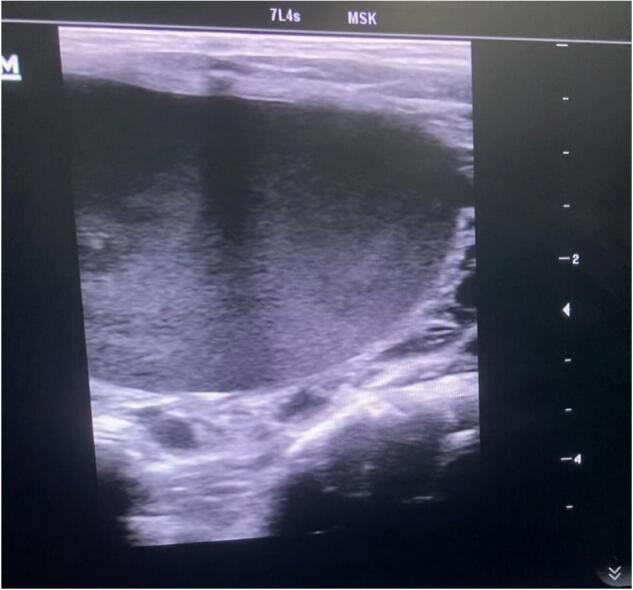
An ultrasound of the neck demonstrated lesion measures about (3.5 × 5 × 6 cm), located at the base of the neck without solid components, which suggests cervical cystic hygroma.

**An ultrasound of the neck** demonstrated multiseptated lesion measures about (3.5 × 5 × 6 cm), located at the base of the neck without solid components, which suggests lymphatic malformation (Fig. 2). Lower aspect of the mass extends retrosternally. Therefore CT was advised for better assessment for lesion extension.

**A computed tomography (CT)** scan with contrast confirmed the presence of a well-defined large cystic mass measures about (4 × 7 × 9 cm), with thin wall and thin septations. No solid components, no calcifications, and no enhancement were noticed. The lesion extends from the left submandibular space inferiorly posterior to the sternocleidomastoid muscle, and retrosternally to the aortic arch level.

It exerts moderate mass effect on the larynx causing a shift to the right and posterior shift to the left carotid sheath components. From the Posterior aspect, the mass reaches the prevertebral space.

**Fig. 3:**
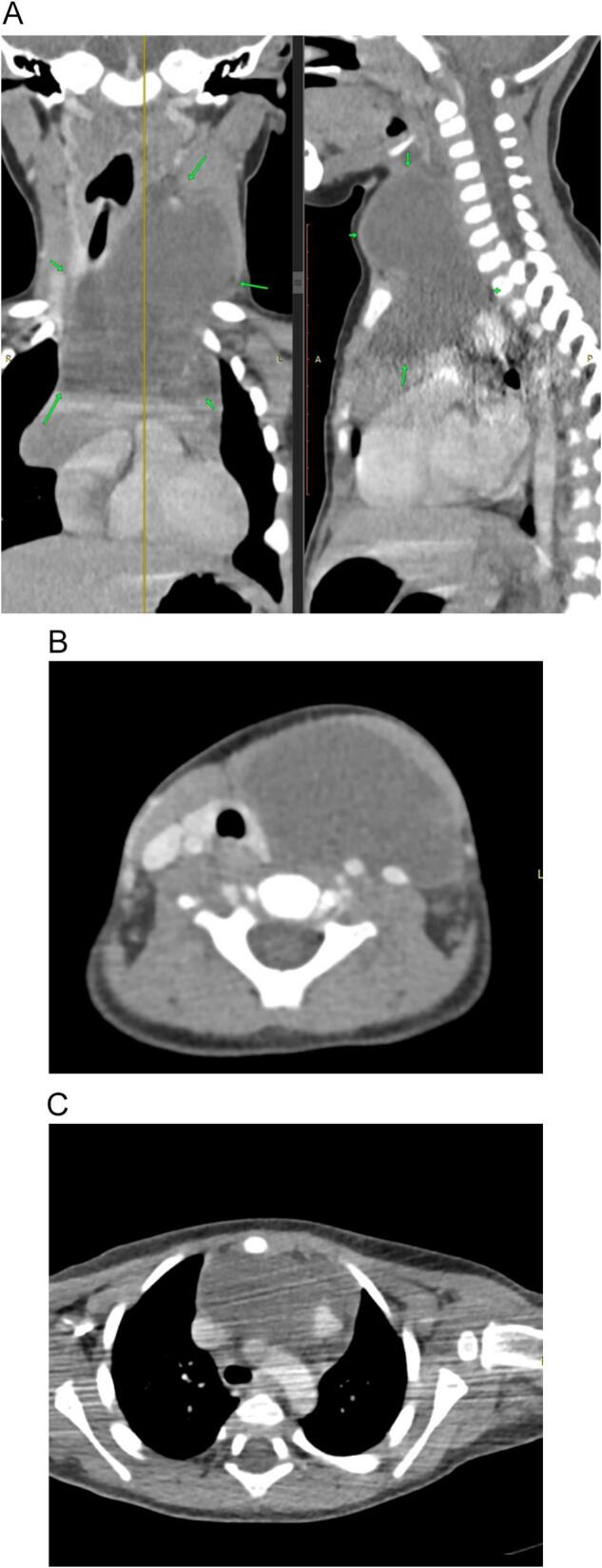
A: CT scan with contrast (sagittal and coronal planes) demonstrate well defined large cystic mass measures about (4 × 7 × 9 cm), with thin wall. The lesion extends from the left submandibular space inferiorly posterior to the sternocleidomastoid muscle, and retrosternally to the aortic arch level. B: CT scan with contrast (axial plane) demonstrates the lesion exerts moderate mass effect on the larynx causing a shift to the right and posterior shift to the left carotid sheath components. From the Posterior aspect, the mass reaches the prevertebral space. C: CT scan with contrast (axial plane) demonstrates retrosternally extension to the aortic arch level.

No lymph adenopathy was noted. No bony erosions. Findings are suggestive of Cystic Higroma (CH) (Fig. 3A–C).

## 5 Therapeutic intervention

According to the staging system proposed by De Serres, the lesion was deemed a macrocytic, stage 3 lesion.

Based on the previous clinical and radiological findings, it was decided to perform Doxycycline sclerotherapy as a primary treatment. The procedure was performed at an operating theatre under general anesthesia. The patient was cleaned and draped. Prophylactic antibiotics were given before the procedure and continued for approximately one week.

**Fig. 4:**
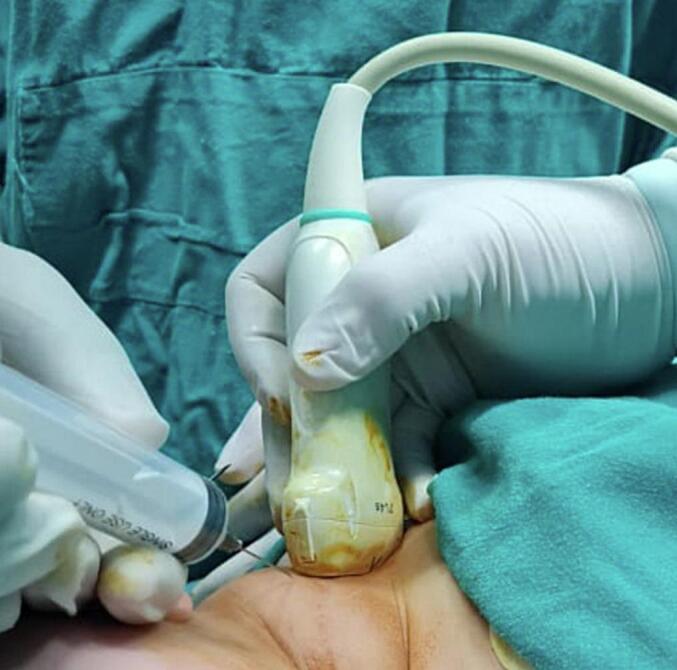
Clinical image showing aspiration of the cystic contents of the lesion using ultrasound guidance.

**Fig. 5:**
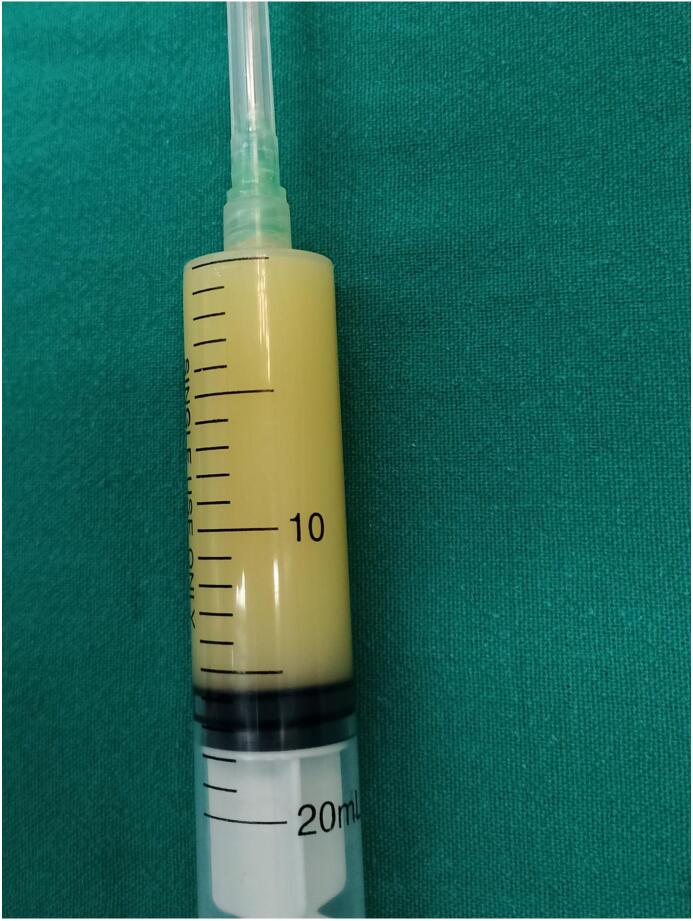
A sample was sent for both microbiological and cytological analysis confirming a sterile cystic hygroma.

Using ultrasound guidance, the macrocysts were cannulated with a 16G IV cannula (Fig. 4), the fluid contents were aspirated as thoroughly as possible. The amount of fluid withdrawn was 60 ml, and a sample (Fig. 5) was sent for both microbiological and cytological analysis confirming a sterile cystic hygroma.

A doxycycline solution was prepared by dissolving 5 capsules of (100 mg of doxycycline) in 50 ml of normal saline. Thus we obtained a solution with a concentration of (10 mg/ml) of doxycycline.

**Fig. 6:**
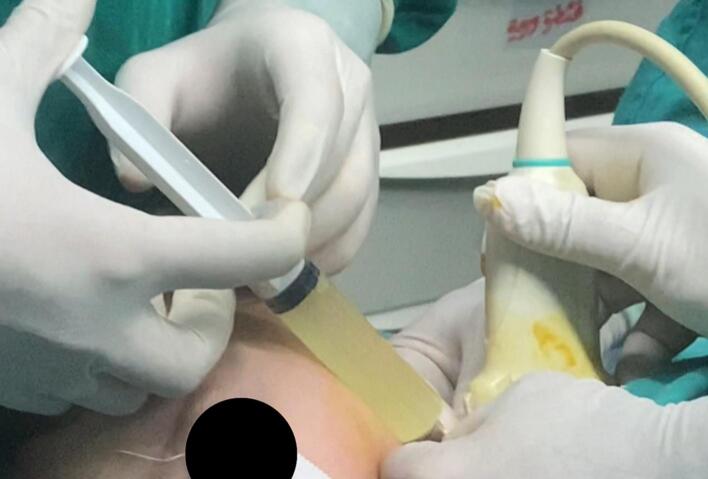
Clinical image showing injection of the doxycycline solution into the lesion.

30 ml of the doxycycline solution was then injected into the pocket of cystic hygroma through percutaneous injection. (The amount of solution injected is equal to half the amount of fluid withdrawn) (Fig. 6).

The patient was left intubated and transferred to the Pediatric Intensive Care Unit, where he was kept sedated for 6 h. Subsequently, the patient was extubated and sent to the ward for 24 h of observation and pain control. There were some local side effects caused by the injection, such as: local erythema, edema at the injection site, and pain. Fortunately improved within 24 h.

**Fig. 7:**
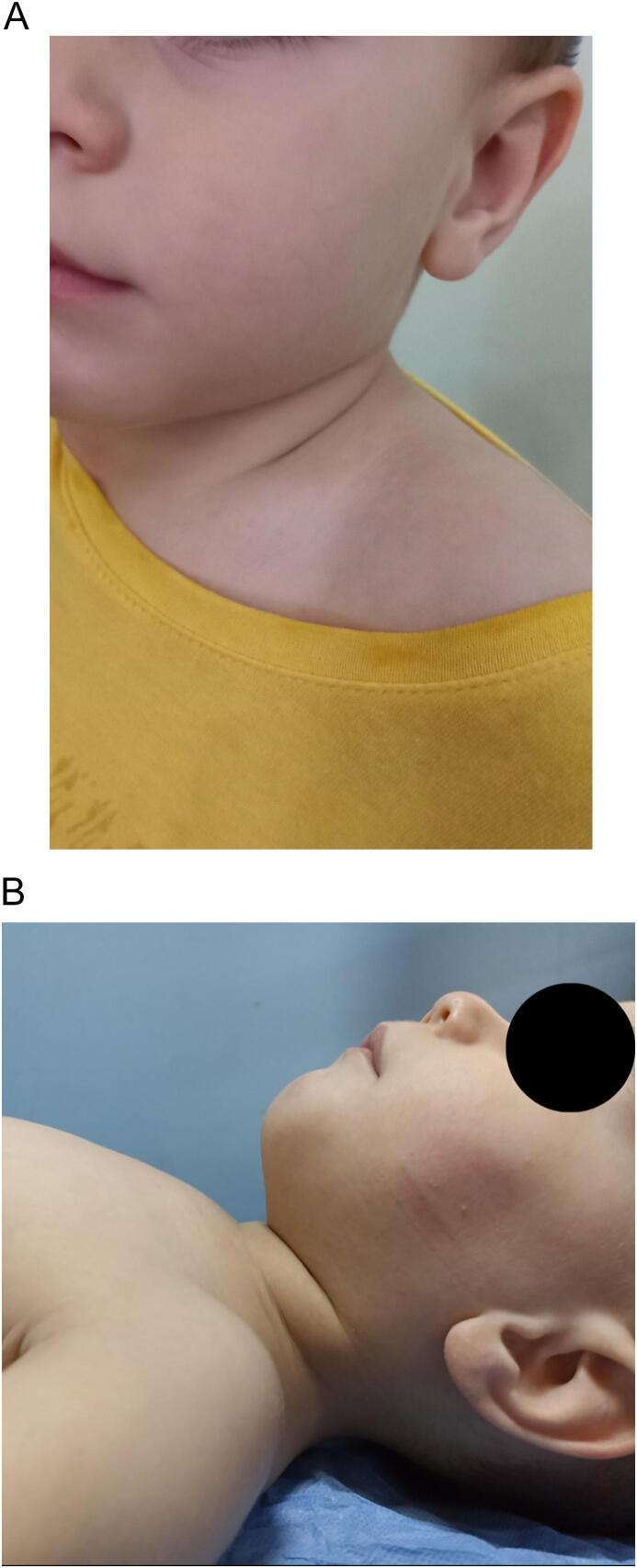
A: Clinical images showing the complete absence of swelling from the left neck observed 12 weeks after the initial procedure. B: Clinical image after 6 months of monitoring demonstrates the lesion did not show any recurrence.

There was a noticeable improvement after 4 weeks and the complete resolution of the cystic hygroma was observed 12 weeks after the initial procedure (Fig. 7A).

During the monitoring period of 6 months, the lesion did not show any recurrence (Fig. 7B).

## 6 Discussion

Cystic hygroma could occur anywhere in the body, but the most common locations are cervical followed by facial and then axilla [1]. Treatment options include watchful waiting, sclerotherapy, surgical resection and combination between them in some cases.

Cystic lymphangiomas are either macrocystic, microcystic, or mixed. Macrocysts were defined as lesions with cystic contents of at least 2 cm3 in volume, whereas microcysts contained lesions less than 2 cm3. Sudden expansion may result from infections or spontaneous intralesional hemorrhage. The response to sclerotherapy depends on the size of the lesion, and fortunately that large cysts respond very well to percutaneous sclerotherapy while patients with microcystic lymphangiomas may not benefit from sclerotherapy [9].

Due to the infiltrative nature of cystic lymphangiomas, complete surgical excision in some instances are unlikely [2]. Incomplete excision eventually leads to lymphorrhea, wound infection, and a high recurrence rate. Moreover, if the tumor wall is thin and fragile, it increases the risk of rupture during the operation [10].

Sclerotherapy which is the alternative, non-invasive treatment (where we inject sclerosing agents) has been found as a safe and effective treatment for CH [11].

There are several advantages of sclerotherapy, like the cosmetic deformity is less common because it requires no incisions and manipulation of overlying skin and soft tissue. In addition, the probability of nerve injury and infections are low.

On the other hand, the delayed effect of sclerotherapy and the need for multiple procedures may be considered disadvantages to this treatment modality.

From this point, in this case, we wanted to apply sclerotherapy only once without repeating it again, and we obtained good and encouraging results through the complete reduction of the lesion during a 6-month of observation without any signs of relapse.

There are a lot of sclerosing agents include bleomycin, ethanol, doxycycline, and OK432 [12,13]. The choice of doxycycline for the index case was based on its availability and safety profile. Historically, it used for pleurodesis of malignant effusions, its effectiveness as a sclerosant for cystic lymphangiomas was first reported by Molitch in 1995. It is believed that doxycycline evokes an inflammatory reaction within the thin-walled cystic lymphangioma, and the resultant effect is involution of the cyst by deposition of collagen and fibrin [14,15].

Therefore, this property was taken advantage of and used in the treatment of (CH) and showed good results. It has also been function as an angiogenesis inhibitor by interfering with cell proliferation and migration via inhibition of matrix metalloproteinase (MMP) and suppression of vascular endothelial growth factor (VEGF)-induced angiogenesis and lymphangiogenesis [15].

The injectable form of doxycycline is also readily available with compounding capability and requires reconstitution in a normal saline solution at a concentration of 10 mg/ml. In addition, doxycycline theoretically may prevent infectious complications. Doxycycline has an established safety profile. Adverse effects associated with doxycycline are reported as local erythema, edema at the injection site, and pain. Mild swelling occurs following injection and typically improves after 24–48 h [16].

There is no standard technique for doxycycline sclerotherapy. Some inject an amount equal to the amount of fluid withdrawn from the cyst and some inject half of the amount withdrawn, and in our case we chose to inject an amount of equal to half the amount withdrawn because the patient is a small child aged 11 months, and according to studies, Doxycycline is known to cause severe discomfort during injection and post procedure for approximately 3 h.

Therefore, sclerotherapy in children is done under general anesthesia and the child was kept intubated and sedated in the PICU for observation for 24 h after the injection, and this is what we did in this case.

Relatively large volumes of doxycycline can be safely used for sclerosis without toxicity as it can be used to treat large cystic lymphangiomas [15] (with a maximum dose of 1000 mg).

A limitation of the study is that it had a short-term follow-up of 6 months. For this reason, long time follow side effects could not be evaluated. Further long-term follow-up studies are needed to assess these.

## 7 Conclusion

Nowadays, surgical excision has become the less popular option for CH with high rates of complication and it has been successfully replaced with intralesional sclerotherapy as approved by many previous studies.

This case report highlights the possibility of safely achieving favorable results with doxycycline sclerotherapy in a developing country.

We encourage the use of this method in the treatment of cyst to be the first treatment option and not surgery due to its ease of application, safety and good availability of the material and in order to avoid the risks associated with surgery that cause complications we do not need.

## Abbreviations

CH: Cystic hygroma

## CRediT authorship contribution statement

**Waddah Al-Saadie**: Otolaryngologist, Conceptualization, resources, who performed the procedure, wrote, original drafted, edited, visualized, validated, literature reviewed the manuscript, and the corresponding author who submitted the paper for publication.

## Consent

Written informed consent was obtained from the patient's parents/legal guardian for publication and any accompanying images. A copy of the written consent is available for review by the Editor-in-Chief of this journal on request.

## Ethical approval

Not required for case reports. Single case reports are exempt from ethical approval.

## Guarantor

Waddah Al-Saadie.

## Research registration number

1. Name of the registry: Doxycycline sclerotherapy as a primary treatment of head and neck giant cystic hygroma: A case report study.
2. Unique identifying number or registration ID: researchregistry10903.
3. Hyperlink to your specific registration: https://www.researchregistry.com/browse-the-registry#home/registrationdetails/675ac885aa7b9e02b55775f5/.

## Provenance and peer review

Not commissioned, externally peer-reviewed.

## Funding

This research did not receive any specific grant from funding agencies in the public, commercial, or not-for-profit sectors.

## Declaration of competing interest

The author declares that there are no conflicts of interest regarding the publication of this paper.

## Data Availability

The datasets generated during and/or analyzed during the current study are not publicly available because the data were obtained from the hospital computer-based in-house system. Data are available from the corresponding author upon reasonable request.
